# Hemadsorption as a Treatment Option for Multisystem Inflammatory Syndrome in Children Associated With COVID-19. A Case Report

**DOI:** 10.3389/fimmu.2021.665824

**Published:** 2021-06-01

**Authors:** Juan Carlos Ruiz-Rodríguez, Luis Chiscano-Camón, Clara Palmada, Adolf Ruiz-Sanmartin, Marina García-de-Acilu, Erika Plata-Menchaca, Janire Perurena-Prieto, Manuel Hernandez-Gonzalez, Marcos Pérez-Carrasco, Pere Soler-Palacin, Ricard Ferrer

**Affiliations:** ^1^ Intensive Care Department, Vall d’Hebron University Hospital, Barcelona, Spain; ^2^ Shock, Organ Dysfunction and Resuscitation Research Group, Vall d’Hebron Research Institute (VHIR), Barcelona, Spain; ^3^ Immunology Division, Vall d’Hebron University Hospital, Barcelona, Spain; ^4^ Diagnostic Immunology Research Group, Vall d’Hebron Research Institute, Barcelona, Spain; ^5^ Pediatric Infectious Diseases and Immunodeficiencies Unit, Vall d’Hebron University Hospital, Vall d’Hebron Research Institute, Universitat Autònoma de Barcelona, Barcelona, Spain

**Keywords:** hemoadsorption, inflammatory multisystemic syndrome, myocardial injury, cytokine - immunological terms, SARS-CoV-2

## Abstract

*Multisystem Inflammatory Syndrome in Children* (MIS-C) *associated with COVID-19* is characterized by hypercytokinemia leading to overwhelming inflammation. We describe the use of a hemadsorption device as part of the supportive treatment for cytokine storm.

## Introduction

There are significant clinical and immunological data describing the clinical consequences of MIS-C as an immunopathogenic illness. Therefore, a key intervention would be the control of the cytokine storm (1–3).

## Case Report/Case Presentation

We present a 17-year-old male with a history of fever, dyspnea, diffuse chest and abdominal pain, and generalized rash on day 13 of COVID-19 molecular diagnosis and within the first two weeks of clinical presentation. The polymerase chain reaction (PCR) test for COVID-19 was negative, while the serum IgG levels were present. The patient presented a rapid deterioration, which evolved into cardiogenic shock with biventricular dysfunction (30% left ventricular ejection fraction on transthoracic echocardiography) and acute hypoxemic respiratory failure. The primary differential diagnoses were sepsis of bacterial origin (including staphylococcal and streptococcal toxic shock syndromes), other systemic viral infections (adenovirus, enterovirus), acute abdomen, Kawasaki disease, drug hypersensitivity, autoimmune or autoinflammatory diseases, and hemophagocytosis.

The blood cultures were negative for bacteria and fungi. The initial evaluations for hemophagocytosis and immunological or pharmacological-related causes were negative. Chest and abdominal CT imaging were performed. The patient had CT findings of ileitis. Blood analyses showed a hyperinflammatory profile (**Table 1**). He required orotracheal intubation and rescue maneuvers for severe hypoxemia. The final diagnosis was narrowed into acute myocarditis secondary to MIS-C (diagnosis of exclusion). The patient fulfilled the Royal College of Pediatrics and Child Health of the United Kingdom (RCPCH-UK) criteria for MIS-C associated with COVID-19 (4). He received empirical treatment with intravenous (IV) amoxicillin-clavulanic, IV methylprednisolone (2mg/kg/day), IV immunoglobulin (1g/kg/d) for three days, and cytokine hemadsorption by a 24-hour treatment with Cytosorb^®^. The patient received the adjunctive therapy with hemoadsorption during the first 24 hours of ICU admission. Informed consent for the initiation of the hemoadsorptive technique was requested from the patient’s family. He required vasopressor and inotropic support that was retired after 4 days of treatment (**Table 1**). Focused bedside transthoracic ultrasound showed progressive improvement of biventricular global function after hemoadsorptive treatment. The patient was discharged from the ICU on day 5 and discharged from the hospital on day 20. In the subsequent outpatient follow-up, the patient has fully recovered to his baseline level of activity, and no chronic sequelae have been detected.

**Table 1:** Evolution of the vasopressor support, clinical anf laboratory values.

	PreHA	PostHA	48h ICU admission	60h ICU admission	Reference values
CRP/ (mg/dL)	30.68	36.25	20.2	19.98	0.03-0.50
Ferritin (ng/mL)	399	619	400	394	25.00-400.0
IL-6 (pg/mL)	3457	47.8	41.92	28.13	0.00-4.30
IL-10 (pg/mL)	90.3	9.24	6.9	7.88	0.00-7.80
CD25s (pg/mL)	16969	16596	12038	14914	400.00-2000.00
DD (ng/mL)	902	1368	603	725	0.00-243.00
SOFA	14	6	2	2	–
P/F	60	400	432	–	–
S/F	–	–	–	326	–
NAD (μg/kg/min)	0.8	0.08	0	0	–
DBT (μg/kg/min)	2	2	0.2	0	–

The hemoadsorption (HA) technique was performed within the first 24h of ICU admission. CRP, C-reactive protein; IL-6, interleukin-6; IL-10, interleukin-10; CD25s, interleukin-2 soluble-receptor; DD, D-dimer; SOFA, Sequential Organ Failure Assessment (points); P/F, PaO_2_/FIO_2_ ratio; S/F, SpO2/FiO_2_ ratio; NAD, noradrenaline; DBT, dobutamine; HA, hemoadsorption (duration 24h).

## Discussion/Conclusion

In critically ill adult patients, hyperinflammation plays a significant role in the pathophysiology of multiple organ failure (5, 6). The clinical and laboratory features of hyperinflammation, timing from SARS-CoV-2 infection onset, and similarities in the disease pattern among adults with COVID-19, support the hypothesis that MIS-C results from immune-mediated injury triggered by SARS-CoV-2 infection (7). Currently, there are clinical, microbiological, and immunological data describing MIS-C as a novel immunopathogenic illness (8, 9).

Several official organizations, such as the Centers for Disease Control of the United States (CDC), the World Health Organization (WHO), or RCPCH-UK, have tried to define the general characteristics of MIS-C. However, there is no consensus regarding case definition and clinical management (10, 11).

Treatment includes IV immunoglobulin (12), high-dose IV corticosteroids, and control of hypercytokinemia. For the control of hypercytokinemia, the use of interleukin-1 (IL-1) (13) antagonist (anakinra) and interleukin 6 (IL-6) receptor antagonist (tocilizumab) (14) has been suggested. Recent studies in SARS-CoV-2 patients have shown that these antagonists do not improve mortality (15–19), and tocilizumab has been associated with an increased risk of nosocomial infections (20). However, these studies have been performed in adults, and their results should not be extrapolated to younger patients, such as ours.

Regarding infection risk, hemoadsorption treatment sessions are of short duration. Thus, the risk of infection of intravascular devices used for extracorporeal support seems low, particularly in critical care units where preventive measures are widely implemented. Short- or mid-term hemoadsorption-associated infections have not been described.

The extracorporeal cytokine hemoadsorption device (Cytosorb ^®^) (21) was approved for its use in critically ill COVID19 patients (22, 23). The cartridge has been previously used for cytokine storm-related hyperinflammatory conditions (24) and has been subject to many recent studies (25). Cytokine storm encompasses a heterogeneous group of disorders characterized by life-threatening hyperinflammation, and may be present in non-infectious pathologies (26). We hypothesized that cytokine removal may ameliorate cytokine storm and provide clinical benefits in our patient´s clinical scenario. Previous experience published in sepsis shows that cytokine hemoadsorption is a safe procedure with no associated adverse effects (27).

In our patient, hemoadsorption achieved a safe and rapid reduction of cytokine levels. From a clinical point of view, fast improvements in shock and multiorgan dysfunction parameters were observed. He had no adverse effects associated with the technique, vasopressor support was reduced by more than 80%, and organic dysfunction, measured by the SOFA score, improved from 14 to 6 points in 24 hours. Cytokine levels decreased considerably, and the rest of the acute phase parameters were progressively reduced in the following hours once the hyperinflammatory stimulus was attenuated. The downstream effects (C-reactive protein, soluble CD25 [sCD25], and ferritin levels) are minimal since it mainly reduces acute phase mediators that can be eliminated by hemoadsorption. However, such mediators are reduced after the attenuation of the cytokine storm. Ferritin as an example, is not removed by hemoadsorption, thus we understand the rise of ferritin as corresponding to the evolution of the base process; the ascent occurs in the first 24 hours and decreased rapidly from this moment on due to the treatment implemented.

As this is a case report, causality cannot be confirmed, particularly when considering the other treatment interventions required (e.g., corticosteroids, immunoglobulins, and multiorgan support). However, there was a close temporal relationship between the initiation of hemoadsorption and the reductions in cytokine levels and clinical improvement. IL-6 and IL-10 levels were significantly reduced in 24 hours. Rapid improvements in respiratory function (PaO_2_/FiO_2_ ratio improved from 60 to 400), shock and organ dysfunction parameters (SOFA score improved from 14 to 6 points) were documented 24 hours from initiation of therapy. The patient´s favorable clinical evolution should be attributed to all therapeutic interventions, though the rapid clinical improvement may be attributed to cytokine elimination by hemoadsorption, as it has not been described as a pharmacological effect of corticosteroids or immunoglobulins. MIS-C requires multifactorial treatment. Although corticosteroids and immunoglobulins could have progressively and ultimately modulated inflammation, we consider hemoadsorption responsible for the rapid improvement in respiratory, hemodynamic, and organ dysfunction. Consequently, we consider hemoadsorption a potential adjunctive therapy in patients with MISC-C severe multiorgan dysfunction. Our results must be confirmed in larger studies.

Our case report has several limitations. We report an isolated clinical case, and a cause-effect relationship cannot be confirmed. However, we are aware of the critical role cytokine storm-control had in the rapid reduction of multiorgan support in our patient, despite having received conventional treatment. Impressively, he was discharged from the ICU on day 5 of admission. The cytokine measurements are those included in our institution´s clinical analyses panel.

Beyond being the first to describe the potential usefulness of hemoadsoption for MIS-C associated with COVID-19, the main strength of this case report relies on the rapid control of hyperinflammation observed by cytokine hemadsorption. In combination with conventional treatment, cytokine hemoadsorption can achieve early clinical improvements. This case report supports the effectiveness of hemoadsorption in hypercytokinemia control, though larger and controlled studies are essential to draw any meaningful conclusion.

## Data Availability Statement

The raw data supporting the conclusions of this article will be made available by the authors, without undue reservation.

## Ethics Statement

We complied with the guidelines for human studies and our research was conducted ethically in accordance with the World Medical Association Declaration of Helsinki. Subject gave their written informed consent to publish their case. Information revealing the subject’s identity is to be avoided.

## Author Contributions

Substantial contributions to the conception or design of the work. Drafting the work or revising it critically for important intellectual content. Revising the manuscript critically for important intellectual content. All authors contributed to the article and approved the submitted version.

## Conflict of Interest

The authors declare that the research was conducted in the absence of any commercial or financial relationships that could be construed as a potential conflict of interest.

## References

[1] Buonsenso D Riitano F Valentini P. Pediatric Inflammatory Multisystem Syndrome Temporally Related With SARS-CoV-2: Immunological Similarities With Acute Rheumatic Fever and Toxic Shock Syndrome. Front Pediatr (2020) 8:574. 10.3389/fped.2020.00574 33042918 PMC7516715

[2] Riphagen S Gomez X Gonzalez-Martinez C Wilkinson N Theocharis P. Hyperinflammatory Shock in Children During COVID-19 Pandemic. Lancet (2020) 395(10237):1607–8. 10.1016/S0140-6736(20)31094-1 PMC7204765 32386565

[3] Carter MJ Fish M Jennings A Doores K Wellman P Seow LJ. Peripheral Immunophenotypes in Children With Multisystem Inflammatory Syndrome Associated With SARS-CoV-2 Infection. Nat Med (2020) 26(11):1701–7. 10.1038/s41591-020-1054-6 32812012

[4] Nijman R Guchtenaere A Koletzko Russell R Copley S Titomanlio L. Peadiatric Infammatory Multisystem Syndrome: Statement by the Peadiatric Section of the European Society for Emergency Medicine and European Academy of Pediatrics. Front Pediatr (2020) 8:490. 10.3389/fped.2020.00490 32984206 PMC7485110

[5] Moore BJB June CH. Cytokine Release Syndrome in Severe COVID-19. Science (2020) 368:473–74. 10.1126/science.abb8925 32303591

[6] Ye Q Wang B Mao J. The Pathogenesis and Treatment of the “Cytokine Storm” in COVID-19. J Infect (2020) 80:607–13. 10.1016/j.jinf.2020.03.037 PMC7194613 32283152

[7] Feldstein LR Rose EB Horwitz SM Collin JP Newhams MM Son MBF. Multisystem Inflammatory Syndrome in US Children and Adolescents. N Engl J Med (2020) 383(4):334–46. 10.1056/NEJMoa2021680 PMC7346765 32598831

[8] Whittaker E Bamford A Kenny J Kaforou M Jones C Shah P. Clinical Characteristics of 58 Children With a Pediatric Inflammatory Multisystem Syndrome Temporallyassociated With SARS-Cov-2. JAMA (2020) 324:259–69. 10.1001/jama.2020.10369 PMC7281356 32511692

[9] Consiglio CR Cotugno N Sardh F Pou C Amodio D Rodriguez L. The Immunology of Multisystem Inflammatory Syndrome in Children With COVID-19. Cell (2020) 183(4):968–81.e7. 10.1016/j.cell.2020.09.016 32966765 PMC7474869

[10] Guidance: Paediatric Multisystem Inflammatory Syndrome Temporally Associated With COVID-19 (2020). Available at: https://www.rcpch.ac.uk/resources/guidancepaediatric-multisystem-inflammatory-syndrome-temporally-associated-covid-19.

[11] Rapid Risk Assessment: Paediatric Inflammatory Multisystem Syndrome and SARS -CoV-2 Infection in Children (2020). Available at: https://www.ecdc.europa.eu/en/publications-data/paediatric-inflammatory-multisystem-syndromeand-sars-cov-2-rapid-risk-assessment.

[12] Sperotto F Friedman KG Son MBF VanderPluym CJ Newburger JW Dionne A. Cardiac Manifestations in SARS-CoV-2-associated Multisystem Inflammatory Syndrome in Children: A Comprehensive Review and Proposed Clinical Approach. Eur J Pediatr (2020) 15:1–16. 10.1007/s00431-020-03766-6 PMC7429125 32803422

[13] Huet T Beaussier H Voisin O Jouveshomme S Dauriat G Lazareth I. Anakinra for Severe Forms of COVID19: A Cohort Study. Lancet Rheumatol (2020) 2:e393–400. 10.1016/S2665-9913(20)30164-8 PMC7259909 32835245

[14] Carter M Shankar-Hari M Tibby S. Pediatric Inflammatory Multisystem Syndrome Temporally Associated With SARS-CoV-2 Infection: An Overview. Intensive Care Med (2020) 14:1–4. 10.1007/s00134-020-06273-2 PMC7556601 33057783

[15] Holt GE Batra M Murthi M Kambali S Santos K Perez Bastidas M. Lack of Tocilizumab Effect on Mortality in COVID19 Patients. Sci Rep (2020) 10:17100. 10.1038/s41598-020-74328-x 33051534 PMC7555891

[16] Kewan T Covut F Al-Jaghbeer M Rose L Gopalakrishan K Akbik B. Tocilizumab for Treatment of Patients With Severe COVID–19: A Retrospective Cohort Study. EClinicalMedicine (2020) 24:100418. 10.1016/j.eclinm.2020.100418 32766537 PMC7305505

[17] Huet T Beaussier H Voisin O Jouveshomme S Dauriat G Lazareth I. Anakinra for Severe Forms of COVID-19: A Cohort Study. Lancet Rheumatol (2020) 2(7):e393–400. 10.1016/S2665-9913(20)30164-8 PMC7259909 32835245

[18] Salama C Han J Yau L Reiss W Kramer B Neidhart J. Tocilizumab in Patients Hospitalized With COVID-19 Pneumonia. N Engl J Med (2020) 384(1):20–30. 10.1056/NEJMoa2030340 33332779 PMC7781101

[19] Stone J Frigault M Serling-Boyd N Fernandes A Harvey L Foulkes A. Efficacy of Tocilizumab in Patients Hospitalized With COVID-19. N Engl J Med (2020) 383:2333–44. 10.1056/NEJMoa2028836 PMC7646626 33085857

[20] Pawar A Desai RJ Solomon DH Santiago Ortiz AJ Gale S Bao M. Risk of Serious Infections in Tocilizumab Versus Other Biologic Drugs in Patients With Rheumatoid Arthritis: A Multidatabase Cohort Study. Ann Rheum Dis (2019) 78(4):456–64. 10.1136/annrheumdis-2018-214367 30679153

[21] Cytosorbents Corporation and CytoSorbents Medical Inc. CytoSorb_ – A First-in-Class Cytokine Filter Approved in the European Union. Available at: www.cytosorbents.com/tech.htm.

[22] U.S. Food and Drug Administration. CytoSorb ® 300 Ml Device Approved by FDA for Emergency Treatment of COVID-19. (2020).

[23] Yang X-H Sun R-H Chen E-Z Liu J Wang H-L Yang R-L. Expert Recommendations on Blood Purification Treatment Protocol for Patients With Severe COVID-19. Chronic Dis Transl Med (2020). 10.1016/j.cdtm.2020.04.002 PMC7186198 32346492

[24] Napp L Bauersachs. Extracorporeal Hemoadsorption: An Option for COVID-19 Associated Cytokine Storm Syndrome. Shock (2020) 18:10. 10.1097/SHK.0000000000001568 PMC7363368 32453251

[25] Al Shareef K Bakouri M. Cytokine Blood Filtration Responses in COVID-19. Blood Purif (2020) 28:1–9. 10.1159/000508278 PMC7490911 32464624

[26] Weaver LK Behrens EM. Weathering the Storm: Improving Therapeutic Interventions for Cytokine Storm Syndromes by Targeting Disease Pathogenesis. Curr Treatm Opt Rheumatol (2017) 3:33–48. 10.1007/s40674-017-0059-x 28944163 PMC5606329

[27] Honore P Hoste E Molnár Z Jacobs R Joannes-Boyau O Malbrain M. Cytokine Removal in Human Septic Shock: Where are We and Where are We Going. Ann Intensive Care (2019) 9:56. 10.1186/s13613-019-0530-y 31089920 PMC6517449

